# Mutation-driven parallel evolution in emergence of ACE2-utilizing sarbecoviruses

**DOI:** 10.3389/fmicb.2023.1118025

**Published:** 2023-02-23

**Authors:** Bin Gao, Shunyi Zhu

**Affiliations:** Group of Peptide Biology and Evolution, State Key Laboratory of Integrated Management of Pest Insects and Rodents, Institute of Zoology, Chinese Academy of Sciences, Beijing, China

**Keywords:** bat virus, SARS-CoV-2, insertion/deletion mutation, point mutation, evolutionary intermediate, functional diversification

## Abstract

Mutation and recombination are two major genetic mechanisms that drive the evolution of viruses. They both exert an interplay during virus evolution, in which mutations provide a first ancestral source of genetic diversity for subsequent recombination. Sarbecoviruses are a group of evolutionarily related β-coronaviruses including human severe acute respiratory syndrome coronavirus (SARS-CoV) and SARS-CoV-2 and a trove of related animal viruses called SARS-like CoVs (SL-CoVs). This group of members either use or not use angiotensin-converting enzyme 2 (ACE2) as their entry receptor, which has been linked to the properties of their spike protein receptor binding domains (RBDs). This raises an outstanding question regarding how ACE2 binding originated within sarbecoviruses. Using a combination of analyses of phylogenies, ancestral sequences, structures, functions and molecular dynamics, we provide evidence in favor of an evolutionary scenario, in which three distinct ancestral RBDs independently developed the ACE2 binding trait *via* parallel amino acid mutations. In this process, evolutionary intermediate RBDs might be firstly formed through loop extensions to offer key functional residues accompanying point mutations to remove energetically unfavorable interactions and to change the dynamics of the functional loops, all required for ACE2 binding. Subsequent optimization in the context of evolutionary intermediates led to the independent emergence of ACE2-binding RBDs in the SARS-CoV and SARS-CoV-2 clades of Asian origin and the clade comprising SL-CoVs of European and African descent. These findings will help enhance our understanding of mutation-driven evolution of sarbecoviruses in their early history.

## Introduction

Coronaviruses (CoVs; *Coronaviridae*, *Nidovirales*) are a group of enveloped, single-stranded, positive-sense RNA viruses with a large RNA genome (~30 kb), comprising four genera (α-, β-, γ-, and δ; Nakagawa et al., 2016; Millet et al., 2021). The 5′-terminal two-thirds of their genomes contain two open reading frames (ORF1a and ORF1ab) coding for replicase polyproteins (pp1a and pp1ab) that are further processed into 16 nonstructural proteins (nsp; Nakagawa et al., 2016). The 3′-terminal one-third of the genome encode structural and accessory proteins. The structural proteins include spike (S), envelope (E), membrane (M), and nucleocapsid (N) proteins, which are required for viral entry, assembly, trafficking, and release of virus particles (Siu et al., 2008; Li, 2016). Of the viral genome-encoding proteins, S protein is the most important determinant of viral infection in that it mediates viral attachment to specific host cell surface receptors and subsequent fusion and viral entry (Hulswit et al., 2016; Li, 2016; Piplani et al., 2021). This protein typically contains ~1,300 amino acids with some sites glycosylated. During viral entry, S protein is cleaved into two distinct structural and functional subunits (S1 and S2) at sites S1/S2 and S2’ (Hulswit et al., 2016; Li, 2016; Piplani et al., 2021). S1 is composed of the N-terminal domain (NTD) and the C-terminal domain (CTD), both used as a receptor-binding domain (RBD) dependent on different viruses (Millet et al., 2021).

**Figure 1:**
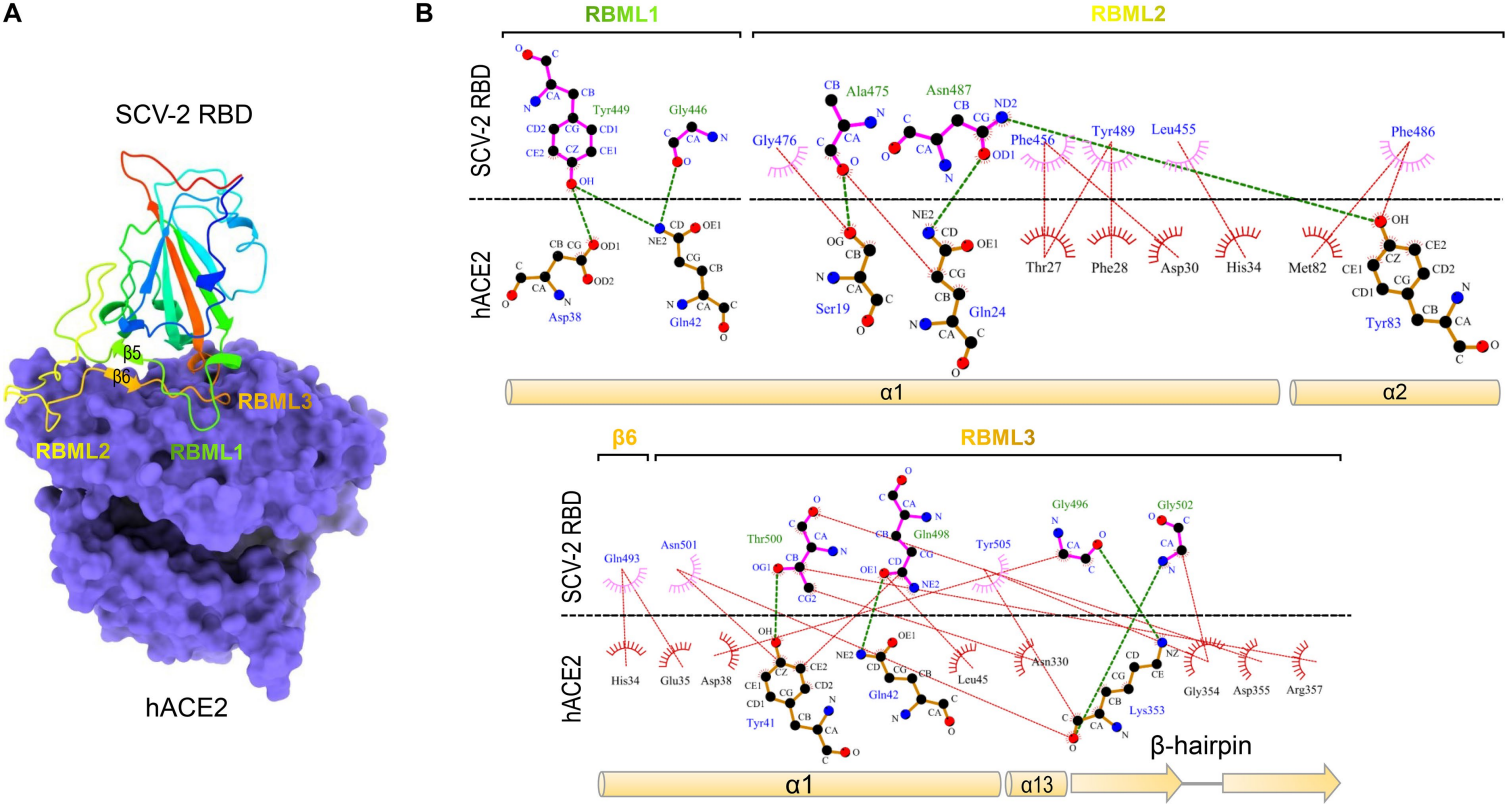
The SARS-CoV-2 RBD binds to hACE2 *via* residues located on the three loops. **(A)** The structure of SARS-CoV-2 RBD and hACE2 complex. The RBM comprising the three loops (designated as RBML1 to RBML3) docks onto the surface of hACE2 (shown in purple; pdb entry 6LZG). **(B)** LigPlot+ plot of the interaction diagram. Hydrophobic contacts and hydrogen bonding between the two loops (RBML1 and RBML2) of the RBD and the two α-helices (α1 and α2) of hACE2 are shown at the top and the interactions between RBML3 and α1, α13, and the β-hairpin of hACE2 at the bottom. The horizontal dotted line represents the interface, in which the residues involved in direct intermolecular hydrophobic contacts are shown as semicircles with radiating spoke and linked by red dotted lines and hydrogens (<4 Å) are represented by green dashed lines.

Severe acute respiratory syndrome coronavirus (SARS-CoV) and SARS-CoV-2 are two highly transmissible and pathogenic β-CoVs that caused serious pandemic in humans (Bolles et al., 2011; Cui et al., 2019; Arya et al., 2021; Harvey et al., 2021). They are two distantly related members of the *Sarbecovirus* subgenus (previously called lineage B) of the genus *β-Coronavirus*. Both viruses likely originated in bats, special reservoirs for emerging zoonotic pathogens (Dobson, 2005; Li et al., 2005a; Brook and Dobson, 2015; Cui et al., 2019; Boni et al., 2020). SARS-CoV and SARS-CoV-2 both use human angiotensin-converting enzyme 2 (ACE2; Kuhn et al., 2004), an enzyme involved in the regulation of cardiovascular and renal function *via* catalysis of angiotensin cleavage (Verano-Braga et al., 2020), as their entry receptor *via* the CTD of their spike protein known as RBD (Li F. et al., 2005; Li et al., 2005b; Hoffmann et al., 2020; Shang et al., 2020; Yan et al., 2020, 2021). The RBD structures of SARS-CoV and SARS-CoV-2 in complexed with human ACE2 (hACE2) have been solved with the aid of X-ray crystallography or cryo-electron microscopy (cryo-EM) techniques (Li F. et al., 2005; Shang et al., 2020). Their molecular cores are highly similar, both containing five anti-parallel β-strands (β1 to β4 and β7) and several short α-helices stabilized by three disulfide bridges (SS1 to SS3; Li F. et al., 2005; Shang et al., 2020). Three loops connect two core β-strands (β4 and β7) and are divided by two anti-parallel β-strands (β5 and β6). They protrude from the core scaffold to assemble a functional unit, named receptor-binding motif (RBM), responsible for direct interactions with hACE2 (Figure 1A). Accordingly, the three loops are, respectively, termed RBML1, RBML2, and RBML3, in which RBML2 is the longest one with one extra disulfide bridge (SS4). The RBM interacts with hACE2 through a large number of hydrophobic and hydrogen-bonding interactions (Figure 1B), in which the loops well match the shape of the highly exposed ACE2 helical regions (Li F. et al., 2005; Shang et al., 2020; Wang et al., 2020).

In addition to these two human viruses, some animal SARS-like CoVs (abbreviated as SL-CoVs) within the Sarbecovirus subgenus can also use ACE2 as their entry receptor, e.g., Rs4084, WIV1 and RaTG13 (Ge et al., 2013; Hu et al., 2017; Li et al., 2021). Their RBDs bind ACE2 with a similar mode to the two human viruses (Liu et al., 2021). Intriguingly, other SL-CoVs closely related to these ACE2-utilizing viruses do not use ACE2 as their receptor (Ren et al., 2008; Ge et al., 2013; Hu et al., 2017, 2018; Roelle et al., 2022). This raises an outstanding evolutionary question regarding how ACE2 binding originated within sarbecoviruses. One opinion thinks that ACE2 binding represents an ancestral and evolvable trait of sarbecoviruses and evolutionary deletions in two specific regions of RBDs led to the loss of the property in the ACE2 non-utilizing SL-CoVs (Shi and Wang, 2011; Starr et al., 2022); the other opinion insists that natural genetic recombination with other evolutionarily related viruses created this property (Boni et al., 2020; Wells et al., 2021). For example, based on phylogenetic reconciliation, it is inferred that extensive ancestral recombination might have occurred in sarbecoviruses including the SARS-CoV-2 lineage (Zaman et al., 2021). Comparative genomic analysis suggests that SARS-CoV-2 may have originated in the recombination of a virus similar to pangolin-CoV with one similar to RaTG13 (Xiao et al., 2020). However, Boni et al. proposed that SARS-CoV-2 itself is not a recombinant of any sarbecoviruses detected to date, and its receptor-binding motif could be an ancestral trait shared with bat viruses and not one acquired recently *via* recombination although the possibility of ancestral recombination events early in the evolution of sarbecoviruses is not excluded (Boni et al., 2020). In these studies, the authors’ points of view are at opposite poles about the role of recombination in the evolution of SARS-CoV-2. Therefore, despite intensive studies worldwide (Hu et al., 2017; Cui et al., 2019; Boni et al., 2020; Xiao et al., 2020; Wells et al., 2021; Zaman et al., 2021), how these sarbecoviruses evolutionarily gained such ability especially in their early history is unresolved and certain to remain controversial, hindering a better understanding of their receptor shift to break through the species barrier.

Mutation and recombination are two major genetic mechanisms that drive the evolution of viruses *via* generating widespread molecular diversity. They both often exert an interplay during virus evolution, in which mutations provide a first ancestral source of functional diversity for subsequent recombination (Arenas et al., 2018). Therefore, although some studies have suggested the role of recombination in the evolutionary gain of ACE2 binding trait in some contemporary sarbecoviruses, it is very likely that mutations have driven the early origin of this trait among the phylogenetically distant ancestral species.

In this study, we employed a combination of analyses of phylogenies, ancestral sequences, structures, functions and molecular dynamics data of the sarbecovirus RBDs and found several key evolutionary events related to ACE2 binding, which had repeatedly occurred in the early evolution of all the three clades of this subgenus, including the SARS-CoV and SARS-CoV-2 clades of Asian origin and the clade comprising SL-CoVs of European and African descent. This suggests that their histories involve parallel evolution on distinct progenitors that ultimately gave rise to the ancestral ACE2-utilizing sarbecoviruses. The proposal of the possible existence of an evolutionary intermediate in the early history of Sarbecovirus evolution will help gain a better understanding of how the viruses gradually evolve to expand their entry mechanisms to enhance their fitness.

## Materials and methods

### LigPlot+ analysis of the RBD-hACE2 complex

For LigPlot+ analysis, hydrogen bonds and hydrophobic interactions were automatically calculated by the HBPLUS program (McDonald and Thornton, 1994; Laskowski and Swindells, 2011) where hydrogen-bond calculation parameters are 2.70 (maximum: H-A distance) to 3.35 (maximum D-A distance; here, H = hydrogen; A = acceptor; D = donor), and non-bonded contact parameters are 2.90 (minimum contact distance) to 3.90 (maximum contact distance). For hydrophobic contacts, hydrophobic atoms are carbon or sulfur. The treatment of connectivity records was used if possible (Laskowski and Swindells, 2011).

### Construction of phylogenetic trees

For constructing the phylogenetic tree of RBDs from the *Sarbecovirus* subgenus, we firstly conducted BLASTP searching against the GenBank database^1^ with SARS-CoV-2 RBD as query to collect homologs and then the retrieved sequences were aligned by ClustalX.^2^ Using this alignment, we inferred a phylogenetic tree by the neighbor joining method with *p* distance to compute the evolutionary distances (NJp method) in the units of the number of amino acid differences per site with MEGA (Yoshida and Nei, 2016)^3^. As a comparison, we also inferred a tree using the Maximum Likelihood method with Whelan And Goldman (WAG) model and a discrete Gamma distribution to model evolutionary rate differences among sites with MEGA, which were chosen by the “Find Best DNA/Protein Model (ML)” mode with the lowest BIC scores (5226.99). Both methods generated similar results with good agreement. For constructing the phylogenetic tree of the whole genomes of the viruses, we conducted BLASTN searching the GenBank database using the full genome sequence of SARS-CoV-2 as query. The retrieved homologs (22 genomes belonging to *Sarbecovirus*; Supplementary Table 1) were aligned with ClustalW implemented in MEGA v10.1.7 (See footnote 3). Using the “Find Best DNA/Protein Model (ML)” model, we analyzed the aligned genome sequences to find the best model of nucleotide substitution for tree construction by maximum likelihood (ML) method. The best model obtained was GTR + G + I with the lowest BIC scores (364135.9), with which we constructed the tree with MEGA. To exam whether a non-*Sarbecovirus* outgroup has a potential impact on the topology of the tree and the evolution direction, we used Middle East respiratory syndrome coronavirus (MERS-CoV; Supplementary Table 1) as outgroup to reconstruct a rooted tree with the same method described above. To exclude the potential impact of RBDs on the whole genome-based tree, we built a sub-genome tree in which all the RBD-coding regions were deleted with the same method described here. The best model obtained was still GTR + G + I with the lowest BIC scores (327206.8). All these trees were built with 500 bootstrap replicates to provide confidence estimates for tree branches.

### Ancestral sequence reconstruction

FastML, a web server for probabilistic reconstruction of ancestral sequences (Ashkenazy et al., 2012), was used to reconstruct ancestral sequences of RBMs of representative sarbecoviruses. This method includes both joint and marginal reconstructions and is especially suitable for the sequences containing indel mutations since it integrates both indels and characters through indel-coding methodology to provide for each indel a presence (‘1’) or absence (‘0’) state in the input sequences. To this end, the amino acid sequences and the genome-based trees with or without the RBD-encoding region were chosen as input files. In this analysis, a discrete gamma distribution was used to account for rate variation among sites and four different evolutionary models of amino acid substitutions (JTT, LG, WAG, and Dayhoff) were chosen to best fits the data analyzed.

### Creation of RBD sequence logo

Two distinct subfamilies of RBDs divided by ClustalX (named RBD-L and RBD-S) were input into the Weblogo server^4^ for creating sequence logos with default parameters. Using the two logos, we calculated the frequency for new amino acid emergence in the RBD-Ls relative to that in the RBD-Ss.

### Preparation of recombinant RBDs

The method for preparation of recombinant SARS-CoV-2 RBD through renaturation from *E. coli*-produced inclusion body (IB) has been reported previously (Gao and Zhu, 2021). According to this method, we produced recombinant proteins of BtRBD derived from the SL-CoV BtKY72 (Protein_id = APO40579.1, residues N^324^ − P^516^) and its mutant BtRBD|GY with two residues (Gly-446 and Tyr-449) inserted in the RBML1. To this end, codon-optimized genes were synthesized from the Tsingke Biotechnology Co., Ltd. (Beijing, China) that were ligated into pET-28a(+) by *Nco* I and *Xho* I restriction enzyme sites with a His tag at both N- and C-termini. Recombinant plasmids were transformed into *E. coli* BL21(DE3) for auto-induction to accumulate IBs under the direction of the T7 promoter. The IBs were then renatured by the previously described method (Gao and Zhu, 2021). Further purification was carried out by size-exclusion chromatography (SEC) with a Superdex™ 75 Increase 10/300 GL column on an AKTA Pure 25 system (GE Healthcare Life Sciences, Pittsburgh, PA, United States) with 1xPBS, pH7.5 as the running buffer and a flow rate of 0.3 ml min^−1^. Peak fractions were pooled and the samples were analyzed by sodium dodecyl sulfate–polyacrylamide gel electrophoresis (SDS-PAGE). Protein concentrations were determined by measuring the absorbance of the protein solution at 280 nm with a UV–VIS Spectrophotometer (NanoDrop2000). The sample was stored at-80°C for use. A Q-TOF mass-spectrometric method was used to determine molecular weights of the purified recombinant RBDs with HPLC-Q-TOF-MS (Agilent Technologies, Chandler, AZ, United States). Recombinant hACE2 (Gln18-Ser740) was purchased from KMD Bioscience (Tianjin, China) which was expressed in HEK293 cells with >95% purity.

### Surface plasmon resonance binding experiments

Surface plasmon resonance (SPR) was used to evaluate the binding of various RBDs to hACE2. The experiments were performed on a Biacore T100 instrument with a CM-5 sensor chip (GE Healthcare Life Sciences, United States) at 25°C according to the method previously described (Zhu et al., 2022). hACE2 was covalently linked on the CM5 sensor chip according to the amine coupling strategy (Nikolovska-Coleska, 2015). For pH scouting procedure, the running buffer used was 1xPBS-T, pH 7.5 with 0.05% Tween 20 and hACE2 was separately solubilized in 10 mM sodium acetate at a final concentration of 25 μg/ml with different pH 4.0, 4.5, and 5.0. For immobilization, the CM5 surface was first activated with two injections of 1-ethyl-3-(3-dimethylaminopropyl)-carbodiimide (EDC 0.4 M) and N-hydroxysuccinimide (NHS 0.1 M; v:v = 1:1) at a flow rate of 10 μl/min and then hACE2 solubilized in 10 mM sodium acetate, pH 4.5 at a final concentration of 25 μg/ml was injected. Non-reacted carboxylic groups on sensor chip surface was blocked by ethanolamine-HCl (1 M, pH 8.5) for 420 s at a flow rate of 10 μl/min. The final immobilization level was 1810 RU.

For detecting binding, an analyte (SARS-CoV-2 RBD, BtRBD or BtRBD|GY) was diluted with the running buffer PBS-T at indicated final concentrations. SARS-CoV-2 RBD was two-fold diluted to final concentrations of 1,000, 500, 250, 125, 62.5, 31.25, and 15.625 nM and BtRBD to final concentrations of 10, 5, 2.5, 1.25, and 0.625 μM. BtRBD-GY was four-fold diluted to final concentration of 40, 10, and 2.5 μM. Diluted samples were injected at a flow rate of 30 μl/min over the immobilized hACE2 during 60 s. Dissociation was monitored for 120 s by injecting the running buffer followed by additional washing for 180 s at a flow rate of 30 μl/min for the completely removal of specifically and non-specifically bound biological material from the surface. Responses were measured in RUs as the difference between active and reference channel. The binding curve was fitted with the software BIAevaluation v2.0.1 using 1:1 Langmuir binding model. The rational of using hACE2 to test the activity of BtRBD and its mutant BtRBD|GY was based on the work of Letko et al., in which the authors used hACE2 as the assay target to evaluate multiple bat-derived SL-CoVs with a long RBD (Letko et al., 2020). They found that many of them were able to use this human receptor for cellular entry (Letko et al., 2020). This experiment confirmed the functional conservation of ACE2 between human and bats, in support of the rational of our experiment.

### Molecular dynamics simulations

**Figure 2:**
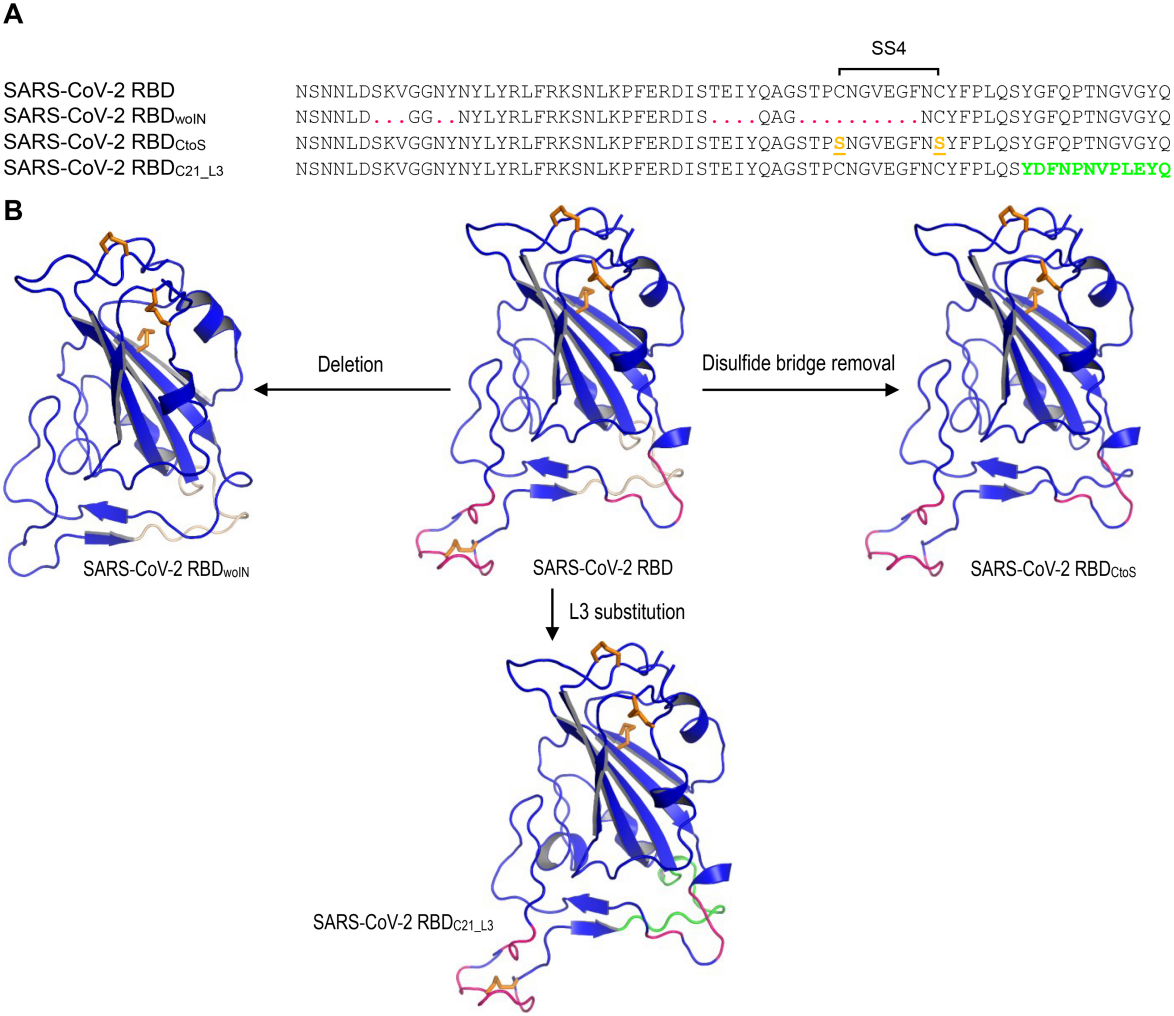
The SARS-CoV-2 RBB mutants for MD simulations. **(A)** The designed RBD mutant sequences. SARS-CoV-2 RBD_woIN_ represents a deletion mutant with corresponding amino acids in RBML1 and RBML2 (marked in red) deleted and “woIN” denotes “without insertions.” SARS-CoV-2 RBD_CtoS_ represents a mutant of two Cys to Ser mutations that remove the SS4 (underlined once and shown in orange). SARS-CoV-2 RBD_C21_L3_ represents a mutant whose RBML3 is substituted by the equivalent of the CoVZXC21 RBD (marked in green). **(B)** Structures of the SARS-CoV-2 RBD mutants generated by homology modelling with SARS-CoV-2 RBD (PDB entry 6LZG) as template.

The structures for MD simulations included: (1) SARS-CoV-2 RBD (PDB entry 6LZG); (2) SARS-CoV-2 RBD_woIN_; (3) SARS-CoV-2 RBD_C21_L3_; (4) SARS-CoV-2 RBD_CtoS_ (Figure 2). The latter three structural models were built by comparative modelling with the DeepView Project Mode at the SWISS-MODEL server,^5^ in which SARS-CoV-2 RBD was used as template. For each structure, a 20-ns MD simulations were performed with the GROMACS 2020.1 software package^5^ using the OPLS (Optimized Potential for Liquid Simulations)-AA/L all-atom force field (2001 aminoacid dihedrals) and TIP3P model for explicit water. Solvent shell thickness was 1.5 nm for the monomers and 3.0 nm for the complex in a cubic box and the total charge of the simulated systems were neutralized by adding sodium or chloride ions. The detailed method has been described previously (Gao and Zhu, 2021). The root mean squared deviation (RMSD) for measuring the difference of simulated structures to the structure present in the minimized, equilibrated system, and Cα root-mean-square-fluctuation (RMSF) that captures the fluctuation for each atom about its average position and gives insight into the flexibility of different structural regions of the simulated protein were calculated with the gmx rms command of GROMACS. In addition, for evaluating the lifetime of the three hydrogen bonds between SARS-CoV-2 RBML1 and hACE2, a 100-ns MD simulations were performed with the method described above except the solvent shell thickness of 3.0 nm instead of 1.5 nm.

### Statistics

Data in Supplementary Figure 1 are expressed as mean ± standard deviation (SD; *n* = 2,001) and statistical significance of means between two groups was determined by one-way analysis of variance (ANOVA) with SPSS Statistics 17.0 (SPSS Inc.).

## Results

### Mutation-driven evolution of RBDs in sarbecoviruses

**Figure 3:**
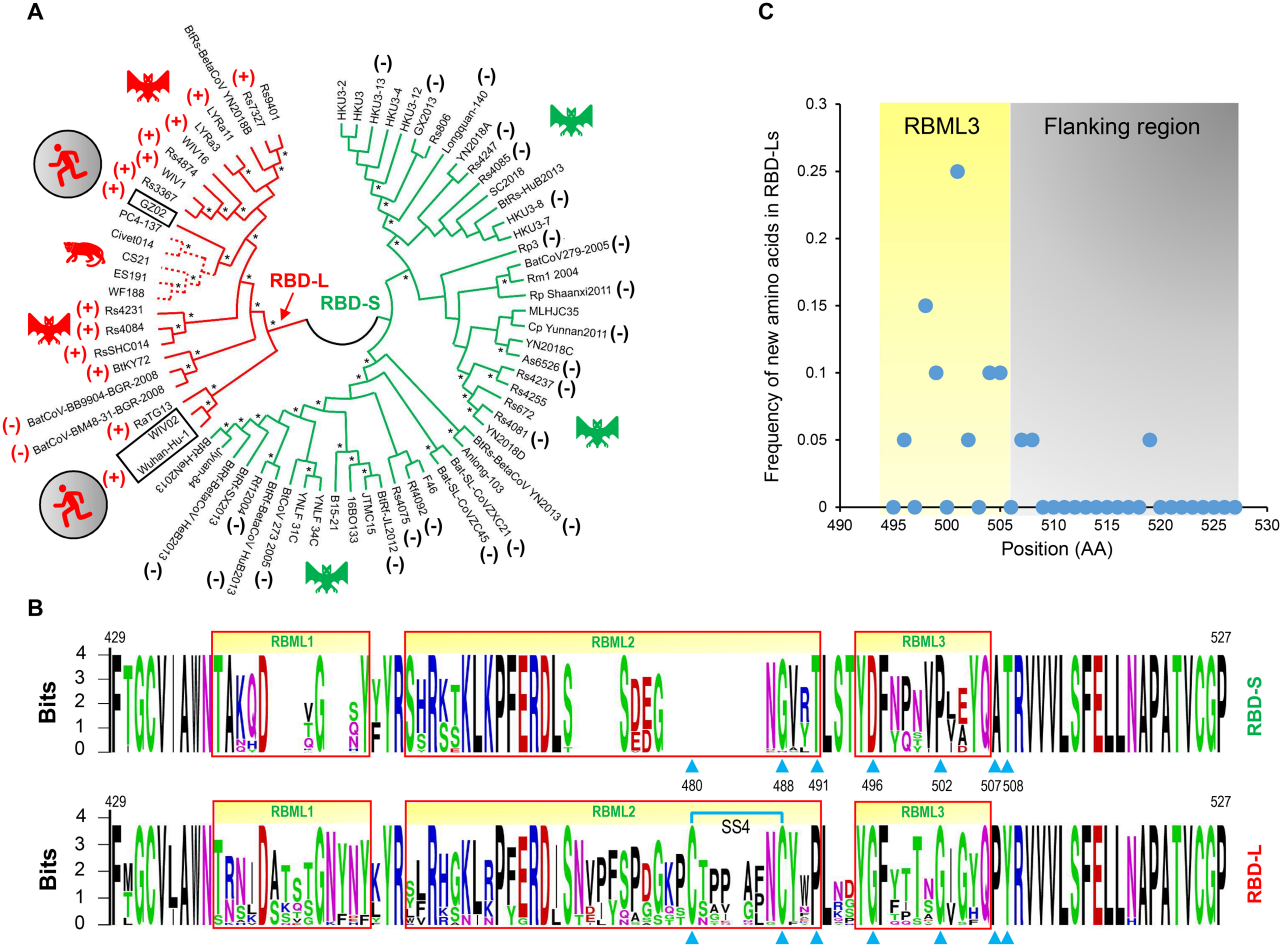
The classification and evolutionary conservation of *Sarbecovirus* RBDs. **(A)** The phylogeny-based classification. The tree was constructed by MEGA with the NJ method based on *p*-distance model of amino acid substitutions (NJp). Asterisks shown at nodes indicate the braches supported by up to 50% bootstrap based on 500 replicates. Two subfamilies differentiated by the tree are denoted as RBD-L (shown in red) and RBD-S (shown in green) based on their loop length. RBDs known to use ACE2 are marked by plus signs and those incapable of binding ACE2 by minus signs (data derived from Ren et al., 2008; Hu et al., 2017, 2018; Letko et al., 2020; Roelle et al., 2022; Starr et al., 2022). Hosts of the viruses, including bats, palm civet and human, are shown here. **(B)** The evolutionary conservation of RBDs analyzed by “Weblogo.” The three loops are boxed in red and positions as tree determinants are indicated by cyan triangles. Top: RBD-S. Bottom: RBD-L. The SS4 in RBD-L is indicated by cyan lines. **(C)** Comparison of the frequency of new amino acids emergence in RBML3 and its flanking region.

Previous studies have found that some bat SL-CoVs with shorter RBML1 and RBML2 in their RBDs are unable to use ACE2 as their entry receptor (Hu et al., 2017), pointing out that the loop length evolution may be related to functional diversification between sarbecoviruse RBDs. To establish a correlation between the loop length and ACE2 binding, we systematically studied a group of RBDs from SARS-CoV, SARS-CoV-2 and SL-CoVs from bats and palm civets, which contained members with both short and long RBMLs (Appendix 1). The mutations considered here included insertion/deletions (indels) altering loop sizes and point mutations altering amino acid sequences. For the uncharacterized RBDs, we used a phylogenomics method to correlate their sequences to the function (ACE2 binding or not). This method overlays known functions onto a phylogenetic tree, on which a sequence’s function can be assigned by its phylogenetic position relative to the characterized ones (Eisen, 1998). To this end, we built a neighbor-joining (NJ) tree based on the amino acid sequences of RBDs (Figure 3A), from which two distinct structural subfamilies were clearly assigned. The ML method yielded a similar tree (Supplementary Figure 2). We named the long RBDs RBD-L and the short ones RBD-S. For the subfamily RBD-L, all members have two extended RBMLs (i.e., RBML1 and RBML2) in length with a 13–18 residues of extension relative to the members from the subfamily RBD-S (Drexler et al., 2010; Tao and Tong, 2019; Letko et al., 2020).

Phylogenomics analyses showed that the tree partitions were overall correlated with the RBD length and their functional properties, in which all members in the RBD-L subfamily are able to bind ACE2 (Ren et al., 2008; Hu et al., 2017, 2018; Letko et al., 2020; Roelle et al., 2022; Starr et al., 2022) except two bat SL-CoVs isolated from Europe (namely BatCoV-BM48-31-BGR-2008, abbreviated as BM48-31, and BatCoV-BB9904-BGR-2008, abbreviated as BB9904; Drexler et al., 2010). The functional loop length of these RBDs is slightly shorter than that of other RBD-Ls (Appendix 1) and the inability of binding to ACE2 has been experimentally confirmed recently in BM48-31 (Letko et al., 2020) and BB9904 (Roelle et al., 2022). For the RBD-S subfamily, all the members are unable to bind ACE2 (Ren et al., 2008; Hu et al., 2017, 2018; Letko et al., 2020; Roelle et al., 2022; Starr et al., 2022; Figure 3A), suggesting that they use an unidentified non-ACE2 receptor in mammals. The overall consistency among the loop indel pattern, the RBD tree topology and functional classification highlights the role of indels in the evolution of ACE2 binding within sarbecoviruses.

We subsequently conducted evolutionary conservation analyses to identify subfamily-specific amino acid positions (Figure 3B). It was found that seven strictly defined positions showed identity within one subfamily but difference in another (Figure 3B), indicating that they are a class of tree determinants that are likely relevant to functional diversification (Valencia and Pazos, 2003). These included Cys-480 and Cys-488 (both forming the SS4), Pro-491, Gly-496, Gly-502, Pro-507, and Tyr-508 in the RBD-L subfamily and the equivalent residues in the RBD-S subfamily are a residue deficiency at 480, Gly-488, Thr-491, Asp-496, Pro-502, Ala-507, and Thr-508 (numbering according to the SARS-CoV-2 RBD; Figure 3B). Because prior studies have shown that in eukaryotic genomes indel mutations often induce an increase in the substitution rate of their flanking regions (Tian et al., 2008; Zhang et al., 2011), we analyzed the frequency of the emergence of new amino acids in the RBML3 of the RBD-L subfamily compared with that of the RBD-S subfamily. The result showed that the RBML3 had a substitution rate of 0.05–0.25 calculated from 20 natural amino acids, which was far higher than that of its flanking region (Figure 3C). These observations suggest that loop extension, tree determining-related point mutations and accelerated substitutions in RBML3 commonly contribute the emergence of ACE2 binding in an ancestral RBD scaffold.

### Structural and functional significance of mutations

**Figure 4:**
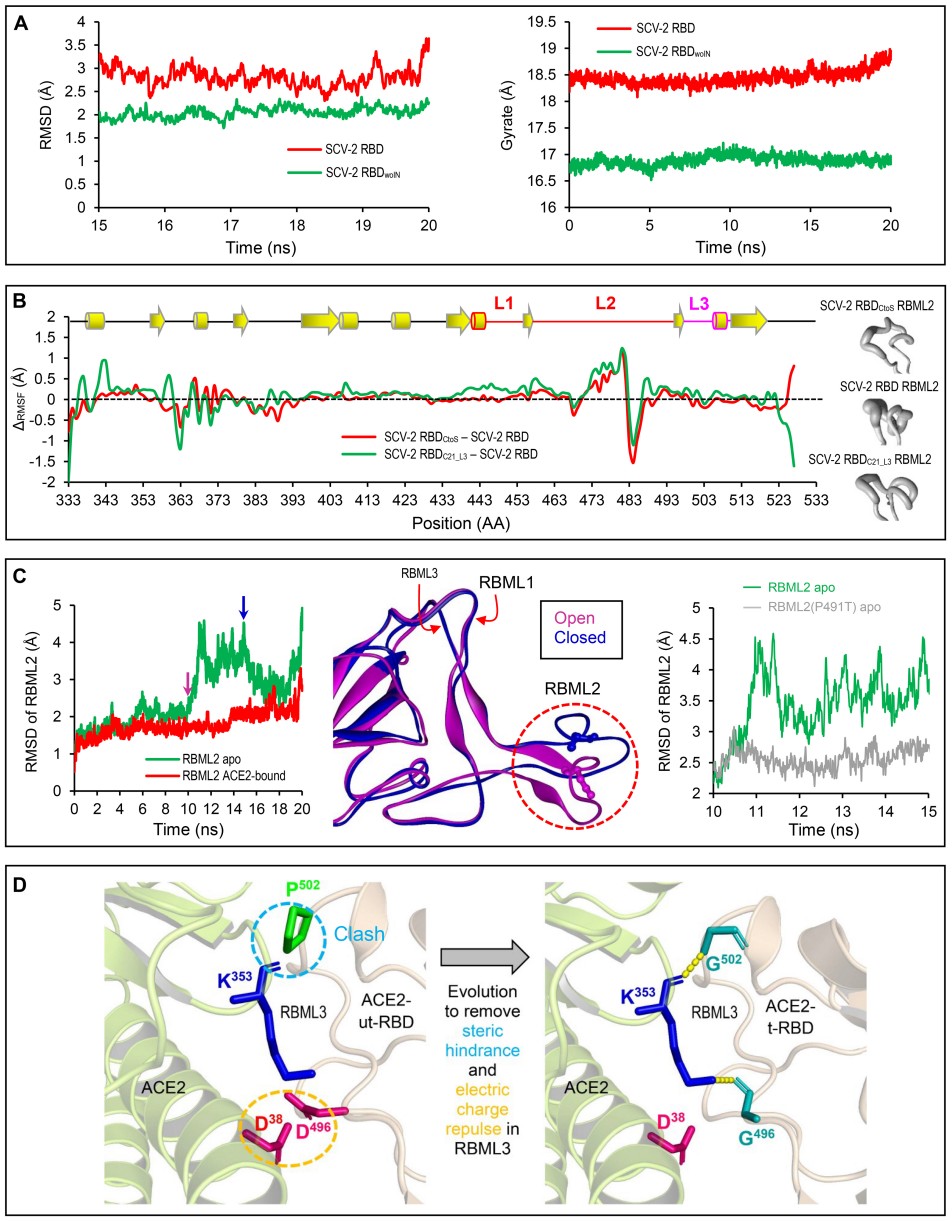
Structural and dynamics evidences for ACE2 binding origin. **(A)** Backbone-RMSDs of SARS-CoV-2 RBD and its deletion mutant shown as a function of time (left). Gyrate of proteins. SARS-CoV-2 RBD and its deletion mutant shown as a function of time (right). **(B)** ΔCα-RMSF data. SARS-CoV-2 RBDCtoS – SARS-CoV-2 RBD is marked in red and SARS-CoV-2 RBDC21_L3 – SARS-CoV-2 RBD in green (left). Conformational ensembles of RBML2 generated by MD simulations and shown by a “sausage” model with MOLMOL (right). **(C)** A 20-ns MD simulations showing structural dynamics of RBML2 in the apo state or ACE2-bound sate (left). Snapshots extracted from the MD trajectories at 10 and 15 ns, respectively, showing two distinct conformations in RBML2 (open and closed; middle). Comparison of the RBML2 RMSDs between SARS-CoV-2 RBD and the P491T mutant (right). **(D)** Structural mapping showing parallel molecular evolution removing steric hindrance and electric charge repulse present in the ancestral state. The clash occurs between Pro-502 of RBDs incapable of binding ACE2 and Lys-353 of ACE2, indicated by a cyan dashed circle, and the electric charge repulse between Asp-496 of the RBDs incapable of binding ACE2 and Asp-496 of ACE2, indicated by an orange dashed circle. In the RBD-hACE2 complex, hydrogen bonds are shown by yellow dashed lines and involved residues displayed as sticks.

To study the potential effects of loop extension and amino acid substitutions on the dynamics of ACE2-binding RBDs, we designed three mutants of the SARS-CoV-2 RBD (Figure 2) for molecular dynamics (MD) simulations. They included: (1) SARS-CoV-2 RBD_woIN_ with RBML1 and RBML2 extensions deleted; (2) SARS-CoV-2 RBD_CtoS_ with two Cys to Ser mutations to remove the SS4 in RBML2; (3) SARS-CoV-2 RBD_C21_L3_ with the RBML3 substituted by the equivalent of the RBD from CoVZXC21, a member belonging to the RBD-S subfamily (Figure 3A). A 20-ns MD simulations revealed that the SARS-CoV-2 RBD exhibited a lower structural stability than the RBD_woIN_, as identified by their RMSD values (~3.0 vs. 2.0 Å) for backbone atoms when calculated in an equilibrium state (15–20 ns; Figure 4A, left). Consistently, the wild-type RBD had a gyration radius of ~18.5 Å greater than that of RBD_woIN_ (~16.9 Å; Figure 4A, right). These data show that the loop extensions in an ancestral RBD incapable of binding ACE2 caused a decrease in the stability of the new molecule but accompanying the emergence of a novel function, indicative of a structure–function trade-off in the RBD evolution, as observed in the evolution of some enzymes, in which they obtained new enzymatic specificities but accompanying the loss of the protein’s stability (Shoichet et al., 1995; Tokuriki et al., 2008).

To examine the effects of the SS4 mutation and the RBML3 substitution (Figure 3C) on the flexibility of different structural regions of RBDs, we calculated their RMSFs for each simulated RBD structures based on the Cα atoms to study the fluctuation degree of the individual amino acids during simulations. By background subtraction of the wild-type RBD RMSF, we found that these two mutations primarily influenced the local flexibility of RBML2 (Figure 4B, left). Consistently, a “sausage” model analysis of the simulated structures showed that this loop in SARS-CoV-2 RBD exhibited greater structural flexibility than that of the two mutants (i.e., RBD_C21_L3_ and RBD_CtoS_; Figure 4B, right). These data suggest that the conformation of RBML2 might be allosterically regulated by mutations at RBML3 (Figures 3C, 4B) in a distant manner or by the evolution of one new disulfide bridge (SS4) in its own region. The former well explains the cause of accelerated substitutions in RBML3 (Figure 3C) when evolved into an ACE2-binding RBD. For the latter, although the prevailing view is that disulfide bridges have been added during evolution to enhance the stability of proteins (Hogg, 2003), it appears that the added SS4 works as a regulator for the conformational flexibility of RBML2.

To study the functional role of loop extensions in ACE2 binding, we compared the dynamics of each loop between the *apo*- (receptor-free system) and ACE2-bound conditions. The time-curves of RMSDs during simulations showed that the RBML1 remained stable in both *apo* and ACE-bound conditions whereas ACE2 binding slightly stabilized the structure of RBML3 (Supplementary Figure 1). Remarkably, RBML2 exhibited a highly conformational flexibility in its *apo* state but ACE binding reduced the flexibility (Figure 4C, left). From the simulation trajectories, we extracted two distinct conformational states (herein named open and closed), in which only the open one is suitable for ACE binding (Figure 4C, middle). Such conformational flexibility may be mediated by Pro-491 because reverse mutation (Pro491Thr) can significantly decrease the flexibility of this loop in the SARS-CoV-2 RBD (Figure 4C, right). Therefore, the location of a proline on the last position of RBML2 (Figure 3) likely acts as a backbone switch controlled by prolyl *cis-trans* isomerization, which allows it to adopt two completely distinct conformations (*cis* and *trans*), as previously documented in other proteins (Schmidpeter and Schmid, 2015).

Among the seven tree determinants recognized here, threes (Cys-480, Cys-488 and Pro-491) have been found to play a potential role in conferring ACE2 binding *via* conformational modulation. Further structural analysis highlights the evolutionary significance of two other tree determinants (D496G and P502G). According to the determined structures of ACE2 complexed with SARS-CoV or SARS-CoV-2 RBD (Li F. et al., 2005; Wang et al., 2020), it can be proposed that the RBD-Ss are energetically unfavorable for ACE2 binding since there exist the electric charge repulse between Asp-496 of these RBDs and Asp-38 of ACE2 and the steric hindrance between Pro-502 of the RBDs and Lys-353 of ACE2 (Figure 4D). Substitutions by introducing a small glycine at these two positions (D496G and P502G) remove the energetically unfavorable interactions and create new H-bonds in the interface (Figures 1B, 4D).

### Phylogenetic evidence for ancestral parallel evolution

**Figure 5:**
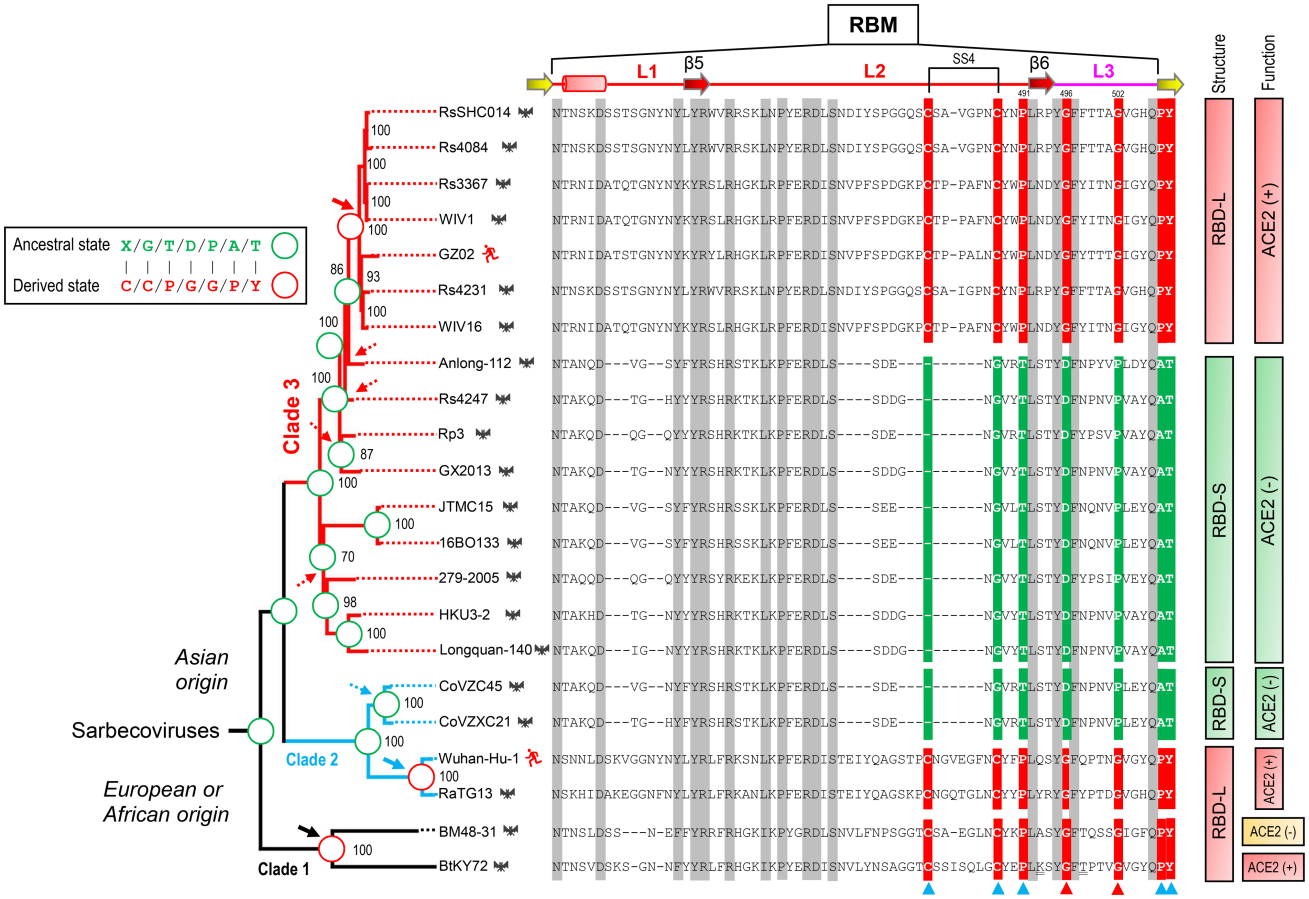
Evidence for parallel evolution of ACE2-binding RBMs in three *Sarbecovirus* clades. Phylogenetic relationships of SARS-CoV (strain GZ02), SARS-CoV-2 (Strain Wuhan-Hu-1) and SL-CoVs were reconstructed based on their whole genomes. The relationships were inferred by using the Maximum Likelihood method with the model GTR + G + I. The percentage of trees (≥70%) in which the associated taxa clustered together is shown at the nodes. Proposed insertion or deletion events are denoted by solid or dotted arrows. Right, sequence alignment of RBML1-3 with conservation across the alignment shaded in grey and the determinants for the topology of RBD-based trees in Figure 2B indicated by triangles, in which two directly associated with functional divergence *via* removing unfavorable interactions are shown in red. Sites involved in parallel substitutions are reconstructed for each nodes, in which the same ancestral amino acids are indicated by green circles and the same derived amino acids evolved through independent changes by red circles. “X” denotes a deficient residue. Structure-and function-based classifications are shown on the right of the alignment: ACE2 (+): RBDs capable of binding ACE2; ACE2 (−): RBDs incapable of binding ACE2 (Ren et al., 2008; Hu et al., 2017, 2018; Letko et al., 2020; Roelle et al., 2022; Starr et al., 2022).

To infer the ancestral state of the mutations related to functional diversification, we reconstructed a phylogenetic tree based on the whole genome sequences of SARS-CoV, SARS-CoV-2 and related SL-CoVs (Figure 5), which is similar to a tree previously published (Lu et al., 2020). We found that adding a non-*Sarbecovirus* outgroup did not substantially alter the topology and the evolution direction of the tree (Supplementary Figure 3). This genome tree is topologically divided into three well supported clades (Figure 5). Clade 1 includes two bat SL-CoVs from Bulgaria and Kenya; clade 2 comprises SARS-CoV-2 and its bat relatives; and clade 3 contains SARS-CoV and its bat relatives. Different from the RBD tree, clades 2 and 3 in this genome tree show no correlation to the indel pattern described above rather than a mixed form of long and short RBDs (Figures 3A, 5). Given new evidence in support of ACE2 binding as an ancestral trait of sarbecoviruses, there are two competitive hypotheses that can be used to explain the histories of the indel mutations in the phylogenetic framework (Figure 5). The first one is three times of independent insertions on three distinct RBD-S-like ancestors which led to the ancestral origins of this trait within sarbecoviruses (Figure 5); the second one is that the common ancestor of sarbecoviruses itself had the insertions and in the subsequent evolution, five times of independent deletions on five distinct RBD-L-like ancestors leading to the loss of the trait (Figure 5). According to the principle of Occam’s razor that entities should not be multiplied unnecessarily (Smith, 1980; Orozco-Sevilla and Coselli, 2020) and for a character evolution the fewest changes are the more likely explanation (Futuyma and Kirkpatrick, 2017), we postulated three times of evolutionary insertions other than five times of deletions more likely mediating the origin of ACE2 binding. Moreover, the deletion hypothesis may require a prerequisite, namely, the common ancestor must possess two receptor entry mechanisms because only this can guarantee their survival when the deletion caused the loss of ACE2 binding. By contrast, our insertion hypothesis does not need this prerequisite. In this case, insertion-mediated loop extension provides key functional residues and structural underpinnings for ACE2 binding, as revealed by their functional importance in ACE2 binding (Figures 1, 4). In the phylogenetic framework, seven amino acid sites previously identified as the tree determinants and their mutations related to ACE2 binding can be defined in two different states: an ancestral state described as X/G/T/D/P/A/T (“X” denoting a deficient residue) and a derived state as C/C/P/G/G/P/Y (Figure 5). This is a typical case of parallel substitutions (Storz, 2016), in which independent changes from the ancestral to the derived occurred three times in evolution. In a sub-genome tree reconstructed based on the genomes with their RBD-coding regions deleted (Supplementary Figure 4), these parallel changes were still retained, indicating that this region does not affect the robustness of the genome tree in exploring the evolutionary events. Again, the deletion hypothesis cannot explain why the parallel substitutions observed here still occur after the loss of ACE2 binding although in a reverse manner.

**Figure 6:**
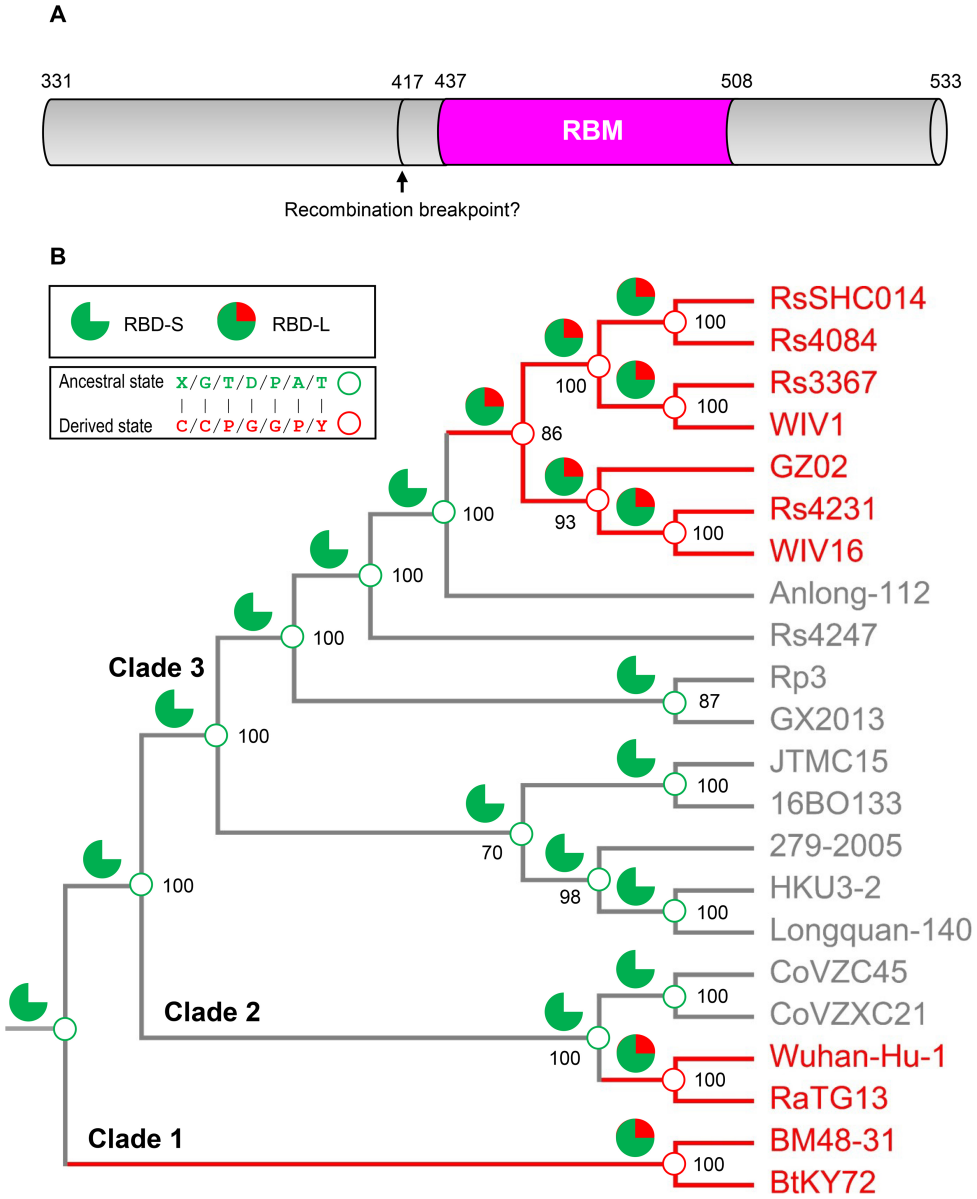
Ancestral sequence reconstruction for elucidating the histories of the insertions and point mutations proposed by parallel evolution. **(A)** Schematic diagram illustrating the structural organization of RBDs, in which the pink region corresponding to the RBM was used to reconstruct ancestral sequences. The putative recombination breakpoint was denoted by an arrow (Starr et al., 2022). Residues were numbered according to the SARS-CoV-2 spike. **(B)** The projection of the ancestral traits including the insertions and point mutations proposed by parallel evolution onto the genome phylogeny. Green sector graphs represent RBD-S without an insertion and green and red sector graphs represent RBD-L with the insertion. Green and red circles represent an ancestral state and a derived state, respectively.

To provide more evidence in support of our hypothesis, we employed an ancestral sequence reconstruction strategy to reconstruct the ancestral states of sarbecovirus evolution with FastML, a method that is especially suitable for the sequences containing indel mutations (see Methods). To minimize the impact of possible recombination, we chose RBM sequences for this end as this region has been predicted to contain no recombination breakpoint (Starr et al., 2022) (Figure 6A). The results show that the ancestral states of the sarbecovirus RBDs (Figure 6B; Supplementary Figure 5) are completely consistent with our hypothesis whatever the genome tree used with or without the RBD-coding region (Figure 6B; Supplementary Figure 4), or different protein substitution models and reconstruction methods used (see Methods). Taken together, our results suggest that the polyphyletic pattern in terms of ACE2 binding in this genome tree is a consequence of ancestral parallel evolution.

### A basal clade-derived RBD incapable of binding hACE2

**Figure 7:**
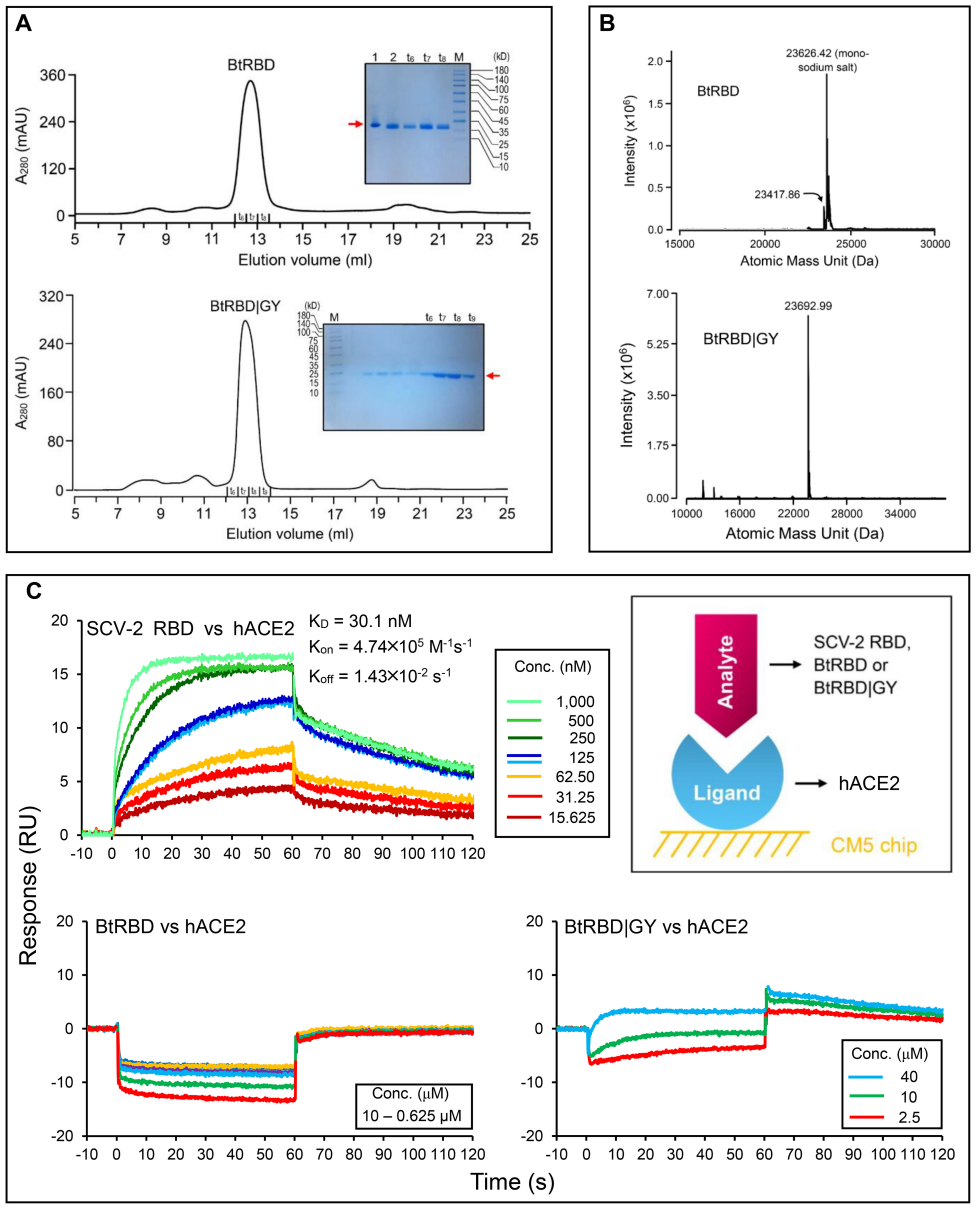
Purification, identification and functional characterization of recombinant RBDs. **(A)** Purification of refolded BtRBD and BtRBD|GY by SEC. Inset: SDS-PAGE analysis of the purified products, marked by a red arrow. “t_6_ to t_9_” denote collection tubes in SEC and “M” denotes protein molecular weight standard. **(B)** HPLC-Q-TOF-MS determining the molecular mass of BtRBD and BtRBD|GY. **(C)** Sensorgrams of SARS-CoV-2 RBD binding to the ACE2-immobilized chip surface (left top). The 125 nM analyte concentration was analyzed in duplicate. The concentrations used were 1,000–15.625 nM with two-fold serial dilutions. Sensorgrams of BtRBD to the chip surface (left bottom). The concentrations used were 10,000–625 nM with two-fold serial dilutions. Sensorgrams of BtRBD|GY to the chip surface (right bottom). The concentrations used were 40,000, 10,000, and 2,500 nM. Inset, schematic diagram of SPR experiment, in which the ligand hACE2 was covalently immobilized onto CM5 *via* its amine groups and the analytes (RBDs) flowed over the surface.

In the genome tree, BtKY72 and BM48-31 are at the base of the radiation of sarbecoviruses and represent the earliest diverged clade of this group (Figures 5, 6; Supplementary Figure 4). Because they occupy a unique phylogenetic position and their RBDs taxonomically fall into the RBD-L subfamily (Figure 3A), we were interested in studying their potential interaction with ACE2. By using the BtKY72 RBD (abbreviated as BtRBD) as a representative, we prepared its recombinant protein through renaturation from *Escherichia coli* inclusion bodies, which was purified by SEC and identified by HPLC-Q-TOF-MS (Figures 7A,B). Subsequently, we employed SPR, a powerful technique for monitoring the affinity and selectivity of biomolecular interactions, to detect its binding with hACE2. SARS-CoV-2 RBD (Gao and Zhu, 2021) was used as the positive control. In this experiment, hACE2 was covalently linked on the CM5 sensor chip and a RBD protein flowed through the chip surface (Figure 7C). The results showed that the SARS-CoV-2 RBD bound to hACE2 with a K_D_ of 30.1 nM [association constant (K_on_) of 4.74 × 10^5^ M^−1^ s^−1^ and dissociation constant (K_off_) of 1.43 × 10^−2^ s^−1^; Figure 7C], which was overall consistent with a previous measurement (Shang et al., 2020). However, BtRBD showed no binding to hACE2 (Figure 7C).

**Figure 8:**
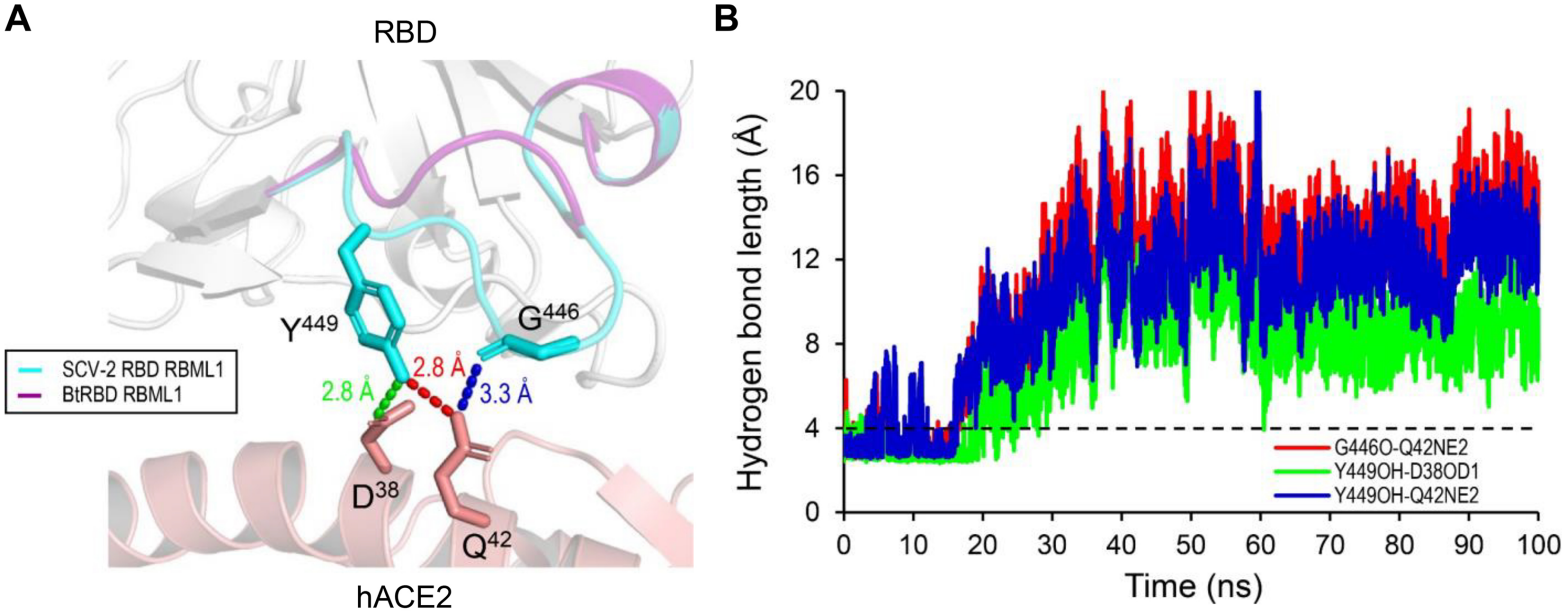
The structural basis of the RBML1 of SARS-CoV-2 RBD interacting with hACE2. **(A)** Gly-446 and Tyr-449 of the RBML1 (colored in cyan) interact with Gln-42 and Asp-38 of hACE2 *via* three hydrogen bonds (pdb entry 6LZG). In the model of BtRBD, its RBML1 far away from the interface is colored in purple. **(B)** The lifetimes of the hydrogen bonds during 100-ns MD simulations. The dashed line represents the length threshold (4 Å) of a hydrogen bond.

Compared with the ACE2-binding RBDs, BtRBD has two deficient residues in its RBML1 (Supplementary Figure 6). These two residues (Gly-446 and Tyr-449) in the SARS-CoV-2 form three hydrogen bonds with hACE2 (Figures 1B, 8A). Due to the deficiency of these two residues, the BtRBD RBML3 was far away from the interface in its structural model (Figure 8A). This provides a possibility to examine their potential effect on hACE2 binding when introduced into the BtRBD backbone. Using the same strategy described above, we prepared this mutant called BtRBD|GY. Unexpectedly, we found that the insertions of these two residues did not evidently improve the binding of BtRBD to hACE2 (Figure 7C). To provide an explanation of this inability, we evaluated the potential functional importance of these hydrogen bonds to the binding of the SARS-CoV-2 RBD to hACE2 *via* MD simulations. In 100-ns simulations, their survival time was smaller than 10% (Figure 8B), suggesting that they belong to a class of short-lifetime hydrogen bonds. Since the contribution of hydrogen bonds to the stability of proteins is strongly context dependent (Pace et al., 2014), we speculated that these hydrogen bonds could only play a secondary role in ACE2 binding. Alternatively, BtRBD and BtRBD|GY might bind bat ACE2 other than hACE2 given its origin from a bat, as reported recently (Starr et al., 2022). In this case, even minimal binding may be sufficient for viral entry, as observed previously in some bat orthologues of hACE2 that could mediate the infection of SARS-CoV and SARS-CoV-2 (Yan et al., 2021). Since the binding and susceptibility are not always consistent, and the ability to support the entry of virus is much more important than the binding in terms of susceptibility to virus infection, more studies to address the significance of the two-residue insertion in BtKY72 infection are needed in the future.

## Discussion

In this work, we have provided multidimensional evidence in support of the role of parallel insertions-and point mutations-driven functional innovation in the ancestral origins of ACE2-utilizing sarbecovirusess. Parallel evolution occurring in multiple evolutionary lineages of viruses are not uncommon (Gutierrez et al., 2019), especially those that register frequent cross-species transmission events (Longdon et al., 2014; Gutierrez et al., 2019). A recent study also showed that the emergence of highly pathogenic avian influenza A viruses is a result of parallel evolution (Escalera-Zamudio et al., 2020). Multiple mechanisms have been proposed to explain such parallel evolution in viruses, such as point mutations involved in the development of antiviral drug resistance, adaptation to new host species, and evasion of host immunity (Gutierrez et al., 2019). For example, a Glu to Lys change at position 627 of PB2 increased virulence on mammalian hosts, in both H5N1 and H3N2 subtypes (Steel et al., 2009). In addition to viruses, parallel evolution has also been documented in animals. For instance, parallel amino acid replacements have resulted in acquired enhanced digestive efficiencies in Asian and African leaf-eating monkeys (Prud'homme and Carroll, 2006; Zhang, 2006). The independent development of closely corresponding adaptive features in two or more groups of mammals that occupy different but equivalent habitats has also been reported previously (Storz, 2016).

Based on the phylogenetic conflict between two trees built from different gene segments, it has been proposed that recombination-mediated exchange of spike RBDs plays a role in the CoV evolution (Boni et al., 2020; Wells et al., 2021). But as mentioned in Introduction, this opinion remains controversial especially in the explanation of the origin of SARS-CoV-2 (Boni et al., 2020; Xiao et al., 2020) and such recombination could not explain how the first ACE2-utilizing sarbecoviruses originated because the RBM directly involved in interaction with ACE2 is not a mosaic organization produced by recombination, as evidenced by the lack of a recombination breakpoint in this region (Starr et al., 2022). We found that for the phylogenetic conflict between the RBD tree and the genome tree, it is more likely explained by parallel evolution-mediated functional clustering of the RBD-L proteins in the RBD tree (Figure 3), which can be recognized by analysis of amino acid changes in the framework of a genome tree and further strengthened by ancestral sequence reconstruction. This well explains the origins of the first ACE2-utilizing sarbecoviruses. The parallel events repeatedly occurred in the evolution of the SARS-CoV and SARS-CoV-2 clades included: (1) Insertion-mediated loop extensions in RBML1 and RBML2. Such extensions created new structural basis through contribution of key structural and functional residues involved in interactions with ACE2 and assembly of one new disulfide bridge modulating the dynamics of RBML2; (2) Insertion-driven substitution rate increase in RBML3 (Figure 3C). These mutations remove energetically unfavorable interactions with ACE2 and affect the dynamics and conformations of the key functional RBML2 (Figure 4). Our observations suggest a role of correlated evolution among different loops in the emergence of ACE2-utilizing sarbecoviruses. Modifications of ancestral loops by molecular tinkering are also in line with the opinion that loops in an ancestral structure are targets for indel mutations during evolution (Pascarella and Argos, 1992).

**Figure 9:**
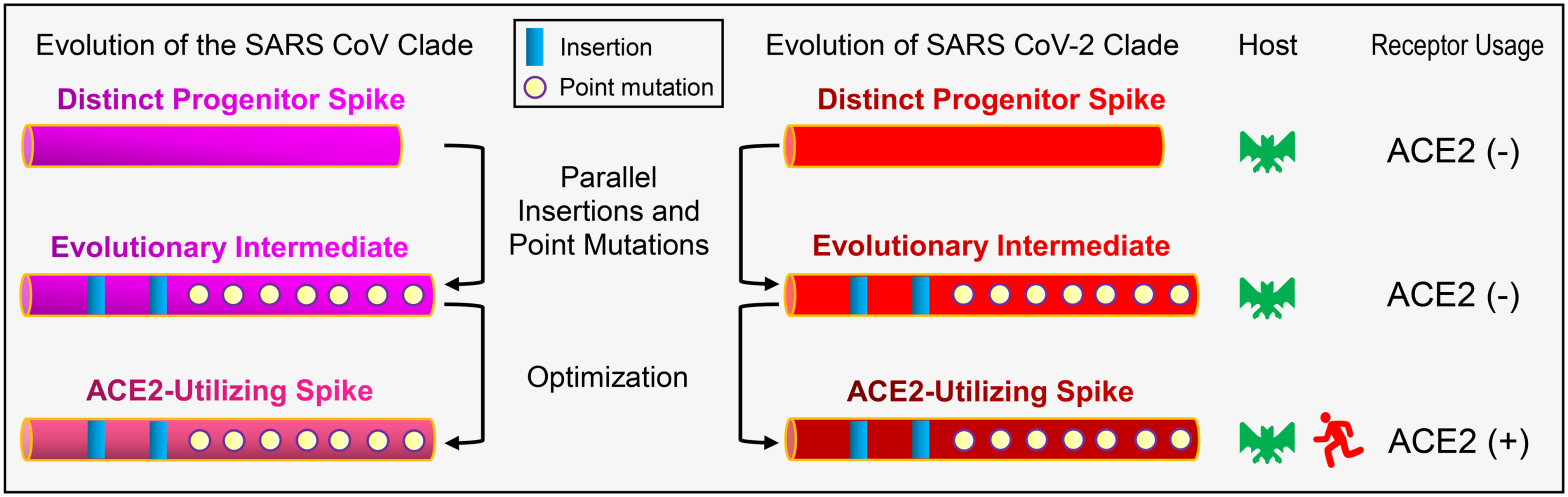
Schematic diagram of the proposed evolutionary histories of ACE2-utilizing spike proteins in sarbecoviruses. For simplicity, only RBDs are shown here. In this evolutionary scenario, two distinct progenitors are developed into SARS-CoV and SARS-CoV-2 clades *via* parallel insertions and point mutations followed by sequence optimization. ACE2 utilizing and non-utilizing are denoted by minus and plus signs, respectively.

Although the events all also occurred in clade 1 (Figures 5, 6), some of its members could not bind ACE2 (Letko et al., 2020; Roelle et al., 2022; Starr et al., 2022). This is likely due to several residues deficiency in the two loops (RBML1 and RBML2), as identified by their length falling between the long and short RBDs. However, adding the deficient residues, as in the case of BtRBD|GY, did not improve the ACE2 binding of BtRBD. It has been found that the development of ACE2 binding on the scaffolds of BM48-31 and short RBDs requires replacing all 14 contact points and the surrounding amino acids in the RBM (Letko et al., 2020). This highlights the role of non-interacting residues in ACE2 binding. During submission of this manuscript, we noticed two recent publications that reported the binding function of BtKY72 RBD to human and bat ACE2 (Roelle et al., 2022; Starr et al., 2022). Our finding that this RBD was unable to bind hACE2 is consistent with (Starr et al., 2022) but different from (Roelle et al., 2022) that recorded some activity on hACE2. Such discrepancy could be due to the difference in the assay methods used (SPR vs. mixed cell pseudotyped virus infection assay; Roelle et al., 2022). Interestingly, this RBD can bind two bat-derived RBDs (Starr et al., 2022). Collectively, these observations suggest that the clade 1 CoVs might represent an evolutionary intermediate linking ACE2 utilizing and non-utilizing sarbecoviruses. We thus propose that parallel evolution in sarbecoviruses could involve a state of evolutionary intermediates (Figure 9). The parallel fixation of key amino acids in these intermediates with different genetic backgrounds might be the first step in an adaptive walk (Storz, 2016) *via* exerting a favorable effect on the mutational pathways of spike protein evolution into ACE2 binding by sequence optimization, as seen in BtRBD whose mutations (K493Y and T498W) enabled it to interact with hACE2 (Starr et al., 2022). If this is true, it suggests that the emergence of ACE2 binding has evolved gradually and repeatedly through molecular tinkering of a pre-existing progenitor over an extended period, as the proposed case for the evolution of the antibody-based immune system (Klein and Nikolaidis, 2005). This suggestion is also highly consistent with the opinion that evolution is often gradual (Futuyma and Kirkpatrick, 2017).

The emergence of a trait from an evolutionary point of view is unlikely to originate more than once by chance and therefore three times of independent origins of ACE2 binding must have been driven by a common selective pressure. Although it is known that viruses and their hosts are locked in an evolutionary arm race (Yap et al., 2020), the fact the ancestral sarbecoviruses still infected bats after they had evolutionarily gained ACE2 binding suggests that the development of the trait is more likely to commonly deal with the insertion-caused decrease in the binding ability of their RBDs to the unknown host receptor other than to circumvent the bats’ defences due to resistance acquirement by the hosts in the arm race. This can be considered as a compensation mechanism during virus evolution and represents an example of mutation-driven evolution of new function (Nei, 2013). A new study provides further support for this opinion. In this study, it was found that the evolutionary gain of an insert in the loop of a nematode defensin leads to the emergence of a new antibacterial function (Gu et al., 2022). Such an insertion event also independently occurred in its ortholog from a genetically distant nematode species (Gu et al., 2018). In particular, our opinion can overall satisfy all four criteria regarding parallel adaptive evolution at the protein sequence level (Zhang, 2006): (1) Similar changes in RBD function occur in three independent evolutionary clades; (2) Parallel amino acid mutations (both insertion and substitution) are observed in these RBDs; (3) A compensation mechanism in receptor usage likely commonly driving their evolution; (4) The parallel mutations are responsible for the parallel emergence of ACE2 binding.

It is worth mentioning that our finding that distantly related coronaviruses independently evolve ACE2 binding in their respective ancestors *via* insertions to increase the flexibility of the functional loop involved in interaction with ACE2, and point mutations to remove unfavorable interactions between RBD and ACE2 is very similar to the evolution of certain toxins. One well-documented example is that insectivorous mammals and lizards both independently evolved their toxins from a class of homologous, ancestral non-toxic enzymes by insertions to increase the flexibility of functional loops and point mutations to introduce new chemical environment (Aminetzach et al., 2009). Also, loop extension and key point mutations were found to jointly drive the emergence of scorpion sodium channel toxins from an ancestral defensin scaffold (Zhu et al., 2020). Although there is no comparability between viral spike proteins and animal toxins, they both may have evolved to use a common strategy to make their weapons.

Different from SARS-CoV and SARS-CoV-2 that both gained receptor binding by parallel evolution to target ACE2, another human coronavirus - MERS-CoV is known to utilize dipeptidyl peptidase 4 (DPP4) instead of ACE2 as the host receptor, which involves the S1 CTD of the spike protein as RBD (Millet et al., 2021). Although these three CoVs all belong to β-coronaviruses and infect humans, the evolutionary mechanisms of their receptor binding origins are different. For MERS-CoV, its spike RBD involved in DPP4 binding (Wang et al., 2013; Xu et al., 2020) exhibits a rather low sequence similarity to the RBDs of other two CoVs involved in ACE2 binding. This could be a consequence of divergent evolution after speciation, which occurred from a common ancestor *via* point mutations and an insertion mutation (Wang et al., 2013; Xu et al., 2020) to target a different host receptor.

Finally, our work highlights the importance of an integrative approach utilizing multidimensional data in exploring the molecular origins of specific phenotypes of viruses from their genotypes. Given that ACE2 is also convergently targeted by HCoV-NL63, a human α-CoV with a similar but distinct ACE2 binding mode from that of β-CoVs (Rawat et al., 2021), our approach is likely to be useful in studying how it originates within the α-CoVs.

## Data availability statement

The original contributions presented in the study are included in the article/Supplementary material, further inquiries can be directed to the corresponding author.

## Author contributions

SZ conceived and designed this study and performed evolutionary analysis and molecular dynamics simulations. BG performed experiments. BG and SZ commonly wrote the paper. All authors contributed to the article and approved the submitted version.

## Conflict of interest

The authors declare that the research was conducted in the absence of any commercial or financial relationships that could be construed as a potential conflict of interest.

## Publisher’s note

All claims expressed in this article are solely those of the authors and do not necessarily represent those of their affiliated organizations, or those of the publisher, the editors and the reviewers. Any product that may be evaluated in this article, or claim that may be made by its manufacturer, is not guaranteed or endorsed by the publisher.

## Footnotes

^1^ https://blast.ncbi.nlm.nih.gov/Blast.cgi

^2^ http://www.clustal.org/

^3^ https://www.megasoftware.net/

^4^ http://weblogo.berkeley.edu/logo.cgi

^5^ https://www.expasy.org/
