# Supplementary material for: Mutation-driven parallel evolution in emergence of ACE2-utilizing sarbecoviruses
Source: Front Microbiol. 2023 Feb 23;14:1118025. doi: 10.3389/fmicb.2023.1118025 (PMC9996049; doi:10.3389/fmicb.2023.1118025)
Supplement: Supplementary file 2 [file Data_Sheet_1.PDF]

Supplementary Material

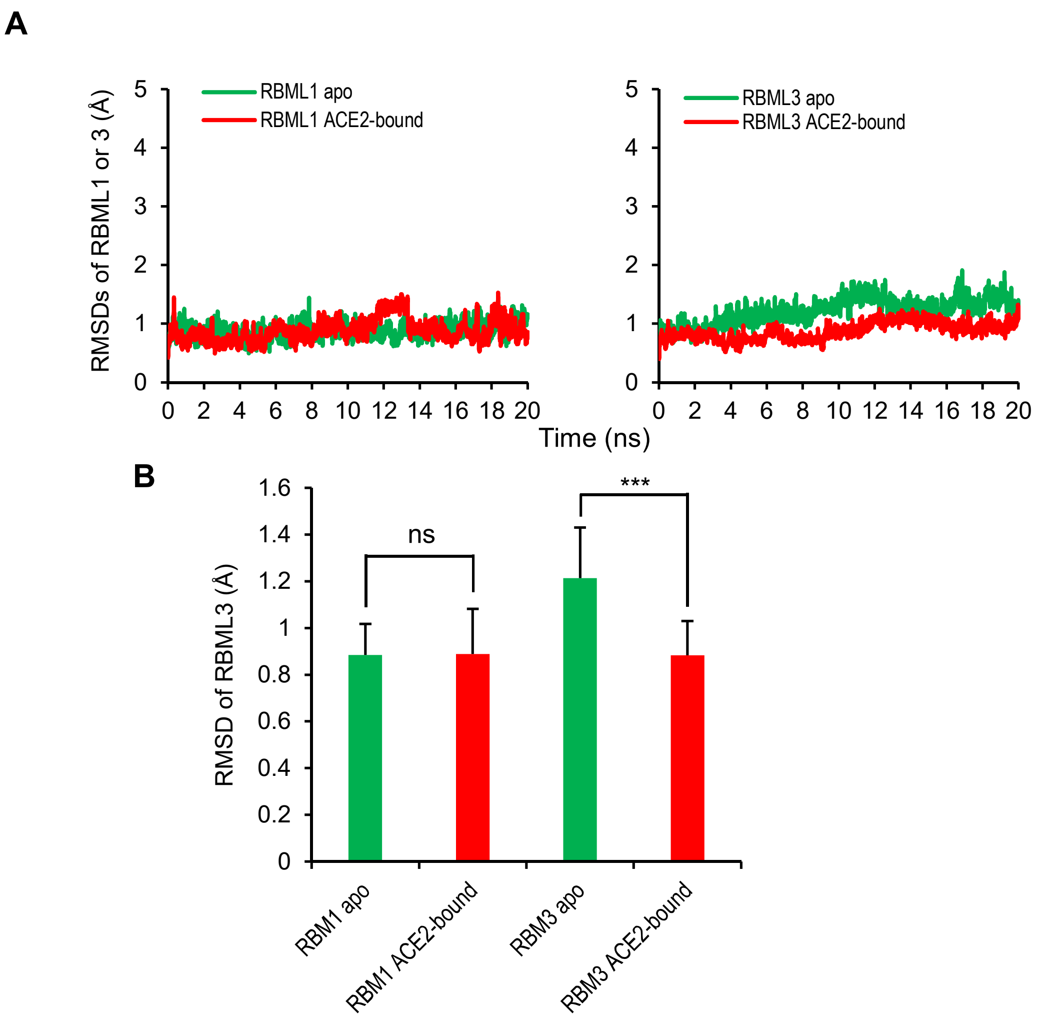

Supplementary Figure 1. The influence of ACE2 binding on the dynamical behavior of RBD loops. (**A**) Comparison of backbone-RMSDs of RBML1 and RBML3 of SARS-CoV-2 RBD in the *apo* or ACE2-bound state shown as a function of time (20 ns simulations). (**B**) Mean ± standard deviation of RMSDs of RBML1 and RBML3 in the *apo* or ACE2-bound state extracted from 20-ns MD simulations (2001 snapshots). "ns", no significance; "***", $P<0.001$.

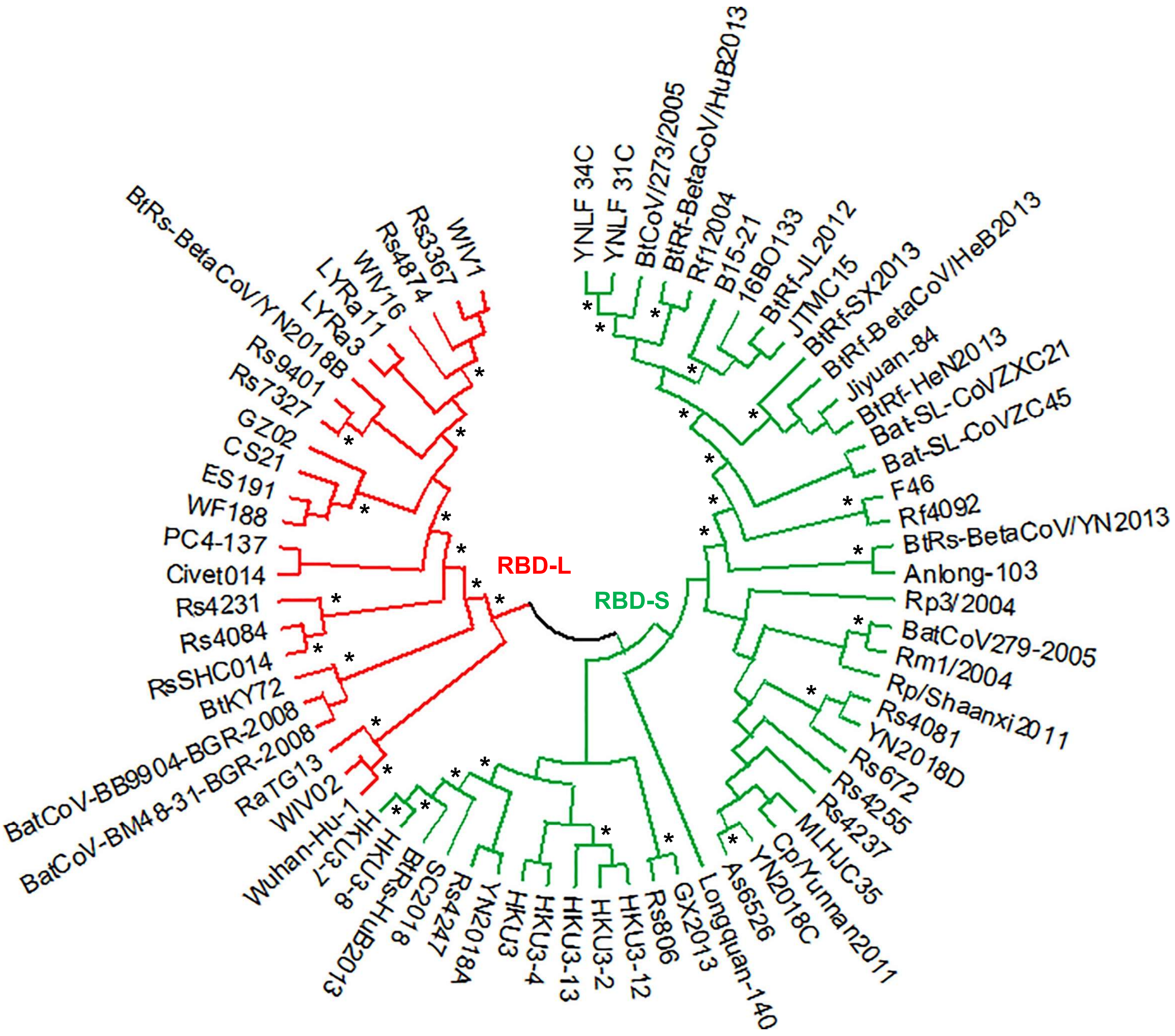

Supplementary Figure 2. The ML tree of the sarbecovirus RBDs. This tree was constructed by MEGA with the model WAG+G. Asterisks shown at nodes indicate the braches supported by up to 50% bootstrap based on 500 replicates. Two subfamilies differentiated by the tree are denoted as RBD-L (shown in red) and RBD-S (shown in green) based on their loop length.

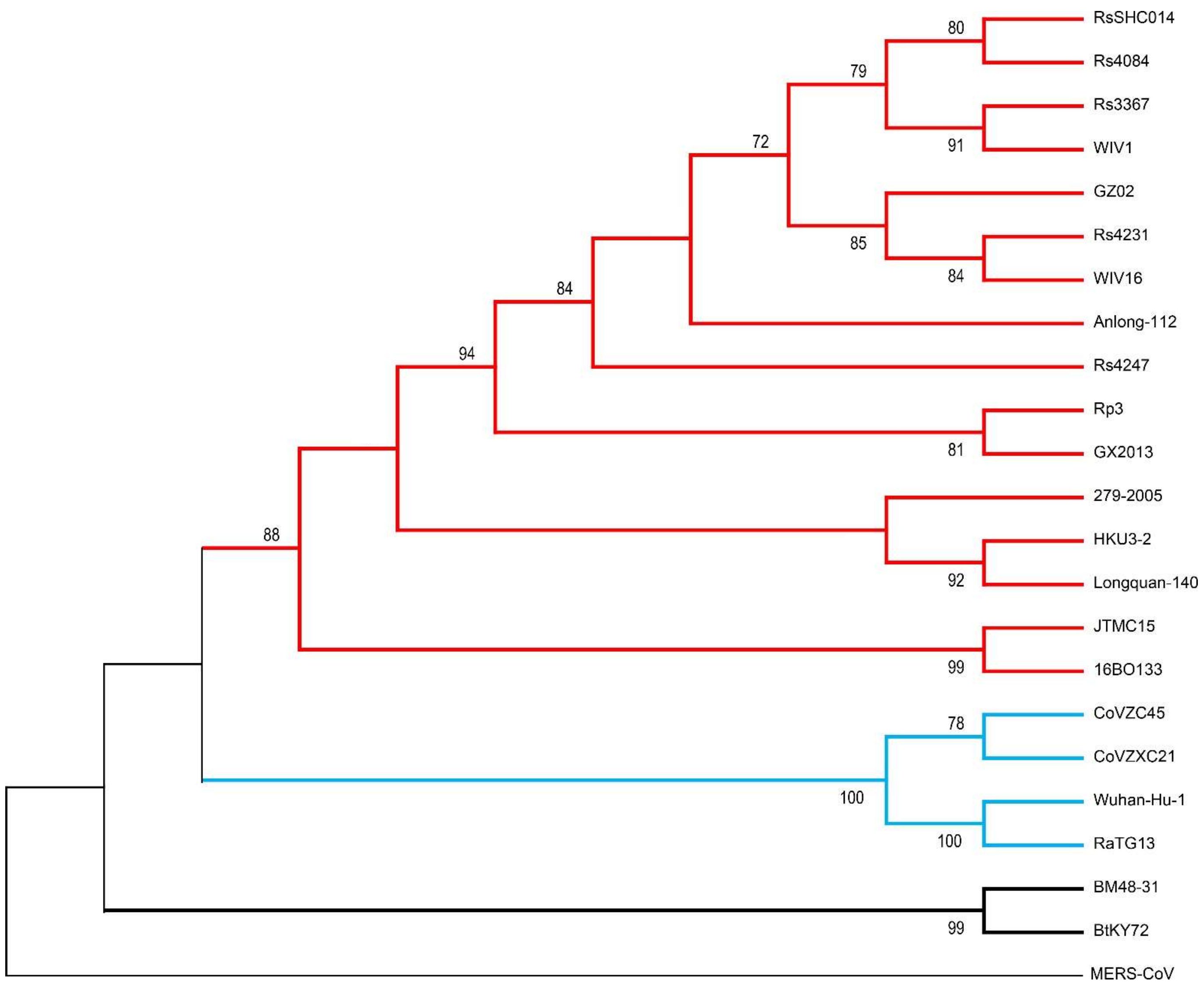

Supplementary Figure 3. A rooted tree revealing the phylogenetic relationships of SARS-CoV (strain GZ02), SARS-CoV-2 (Strain Wuhan-Hu-1) and SL-CoVs. The tree was reconstructed based on the whole genomes. The relationships were inferred by using the Maximum Likelihood method with the model GTR+G. The percentage of trees (≥70%) in which the associated taxa clustered together is shown at the nodes. MERS-CoV was used as an outgroup. Three distinct clades are shown in different colors.

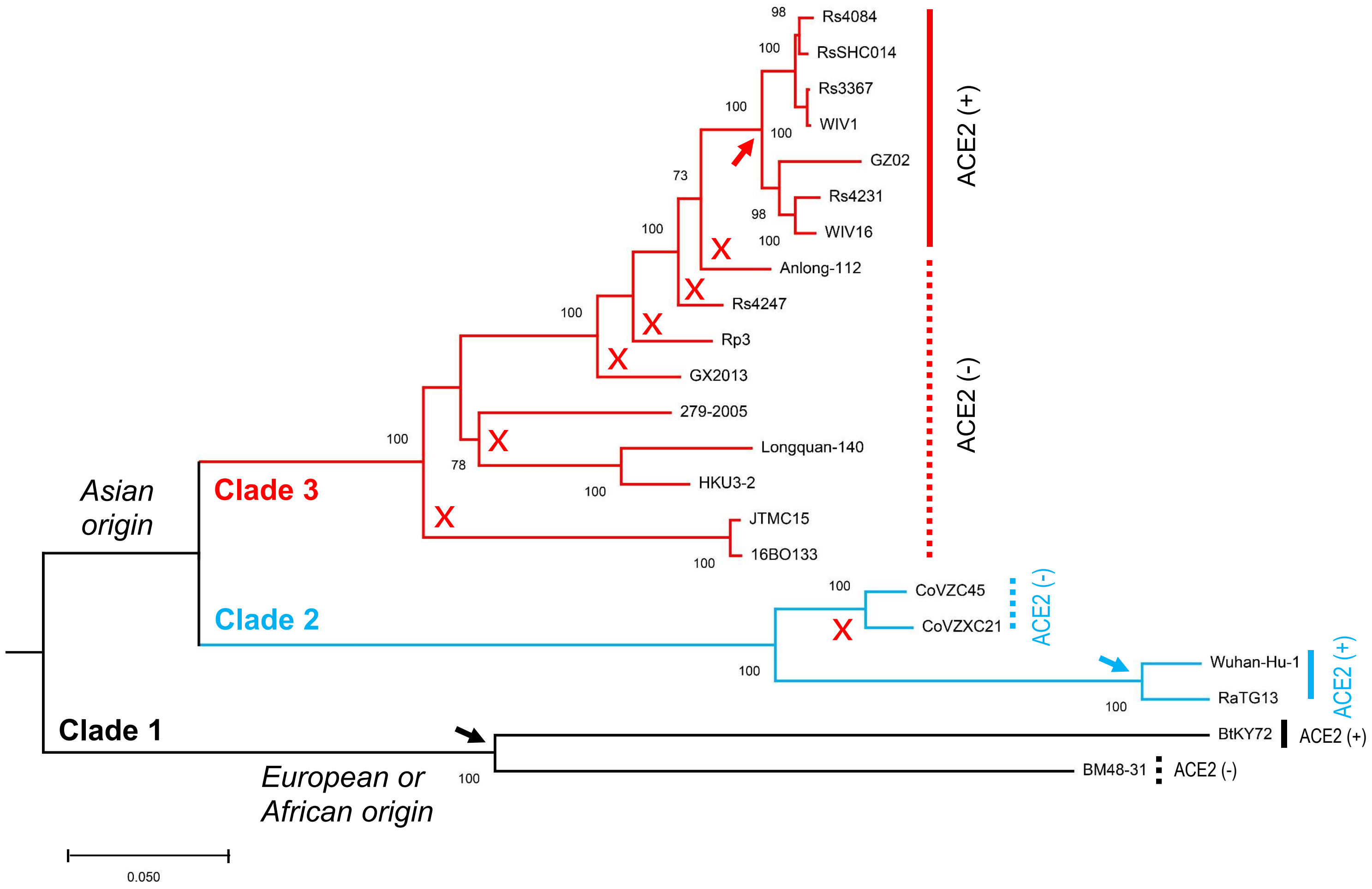

Supplementary Figure 4. Phylogenetic relationships of SARS-CoV (strain GZ02), SARS-CoV-2 (Strain Wuhan-Hu-1) and SL-CoVs. This tree was reconstructed based on their whole genomes with the RBD-coding region deleted. The relationships were inferred by using the Maximum Likelihood method with the model GTR+G+I. The percentage of trees (≥70%) in which the associated taxa clustered together is shown next to the branches. Proposed insertion or deletion events are denoted by solid arrows or crosses. ACE2 binding (+) or not (-) is shown on the right of the tree. The tree is drawn to scale, with branch lengths measured in the number of substitutions per site.

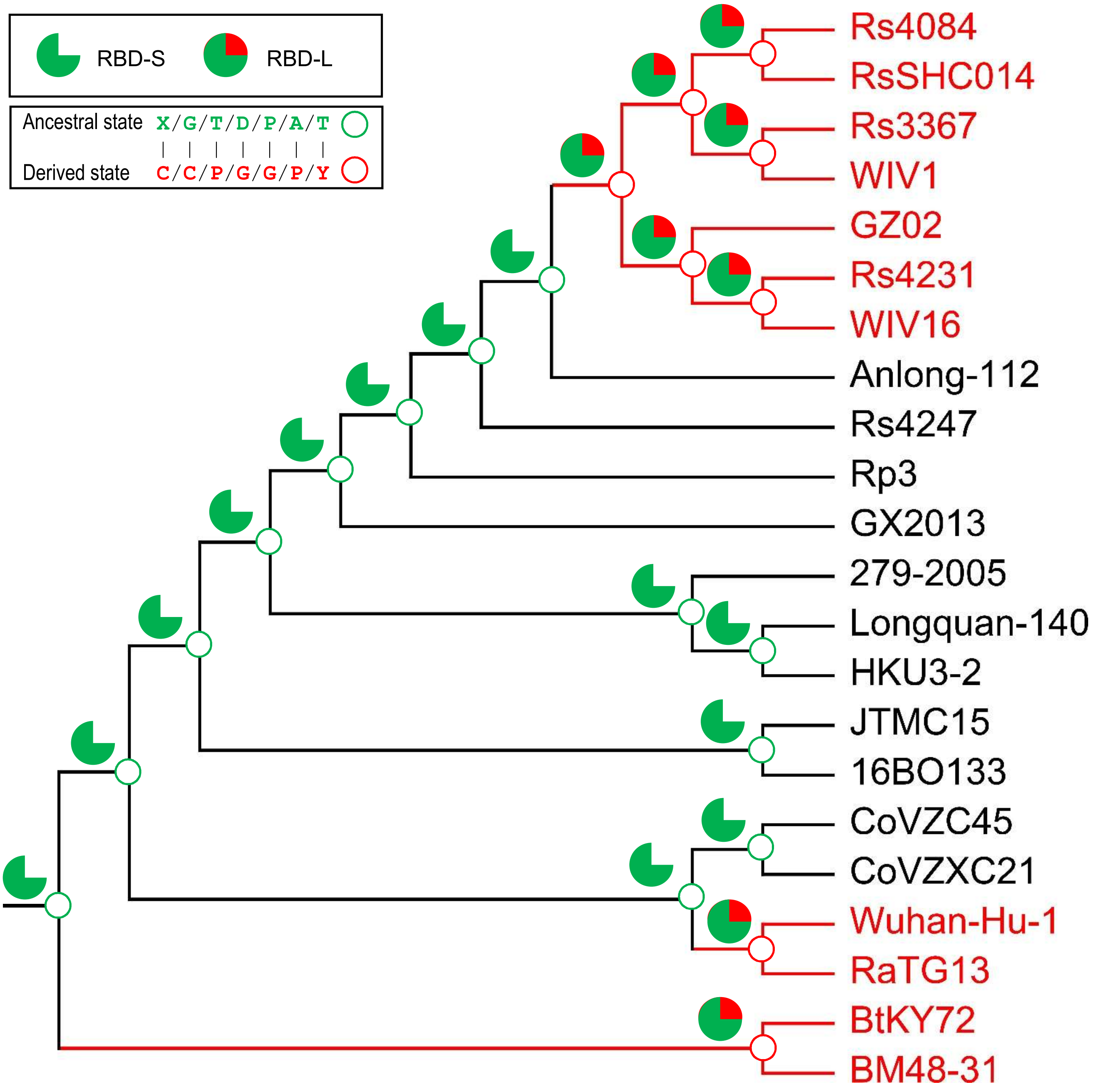

Supplementary Figure 5. The projection of the ancestral traits onto the phylogeny. The tree was built from the whole genomes with the RBD-coding region deleted and the traits include the insertions and point mutations proposed by parallel evolution. Green sector graphs represent RBD-S without an insertion and green and red sector graphs represent RBD-L with the insertion. Green and red circles represent an ancestral state and a derived state, respectively.

	438						445						451
SARS-CoV-2 RBD	S	N	N	L	D	S	K	V	G	G	N	Y	N	Y
BtRBD	T	N	S	V	D	S	K	S	–	G	N	–	N	F
BtRBD|GY	T	N	S	V	D	S	K	S	G	G	N	Y	N	F

Supplementary Figure 6. Sequence comparison of RBML1 between SARS-CoV-2 RBD and BtRBD. The deficient residues in BtRBD are indicated by "-" and the inserted ones in BtRBD|GY are shown in red (numbering according to SARS-CoV-2 RBD). Identical residues are shaded in yellow.

Supplementary Table 1. The sequence accession numbers for the viral genomes used in this study

Viral strain	Accession number
RsSHC014	KC881005.1
Rs4084	KY417144.1
Rs3367	KC881006.1
WIV1	KF367457.1
GZ02	AY390556.1
Rs4231	KY417146.1
WIV16	KT444582.1
Anlong-112	KY770859.1
Rs4247	KY417148.1
Rp3	DQ071615.1
GX2013	KJ473815.1
JTMC15	KU182964.1
16BO133	KY938558.1
279-2005	DQ648857.1
HKU3-2	DQ084199.1
Longquan-140	KF294457.1
CoVZC45	MG772933.1
CoVZXC21	MG772934.1
Wuhan-Hu-1	NC_045512.2
RaTG13	EPI_ISL_402131
BM48-31	NC_014470.1
BtKY72	KY352407.1
MERS-CoV	NC_019843.3
